# Decoding the Heart through Next Generation Sequencing Approaches

**DOI:** 10.3390/genes9060289

**Published:** 2018-06-07

**Authors:** Michal Pawlak, Katarzyna Niescierowicz, Cecilia Lanny Winata

**Affiliations:** 1 International Institute of Molecular and Cell Biology in Warsaw, 02-109 Warsaw, Poland; mpawlak@iimcb.gov.pl (M.P.); kniescierowicz@iimcb.gov.pl (K.N.); 2 Max-Planck Institute for Heart and Lung Research, 61231 Bad Nauheim, Germany

**Keywords:** next generation sequencing, genomics, epigenetics, whole exome sequencing, RNA-seq, ChIP-seq, heart development, congenital heart disease, heart regeneration, GWAS

## Abstract

Vertebrate organs develop through a complex process which involves interaction between multiple signaling pathways at the molecular, cell, and tissue levels. Heart development is an example of such complex process which, when disrupted, results in congenital heart disease (CHD). This complexity necessitates a holistic approach which allows the visualization of genome-wide interaction networks, as opposed to assessment of limited subsets of factors. Genomics offers a powerful solution to address the problem of biological complexity by enabling the observation of molecular processes at a genome-wide scale. The emergence of next generation sequencing (NGS) technology has facilitated the expansion of genomics, increasing its output capacity and applicability in various biological disciplines. The application of NGS in various aspects of heart biology has resulted in new discoveries, generating novel insights into this field of study. Here we review the contributions of NGS technology into the understanding of heart development and its disruption reflected in CHD and discuss how emerging NGS based methodologies can contribute to the further understanding of heart repair.

## 1. Introduction

The vertebrate heart develops through a complex process which involves the interaction between multiple factors at the molecular, cell, and tissue levels. The heart muscle or myocardium comprises the majority of the heart tissue and is mainly responsible for its contractile function. Heart muscle cells or cardiomyocytes (CMs) are specified early in development from a pool of mesodermal progenitors located at the anterior portion of the embryonic lateral plate mesoderm [1]. As development progresses, these progenitors migrate to the embryonic midline, forming a tube structure known as the primitive heart tube which subsequently expands, mainly through cell division. This heart tube subsequently undergoes looping which gives rise to the heart chambers. Although the heart of different vertebrate species can have between two to four chambers, the stepwise process of morphogenesis is highly conserved at the molecular level [2]. Each of the processes of heart morphogenesis is orchestrated by a wide range of regulatory proteins including transcription factors (TFs) and signaling proteins. In addition to this, epigenetic factors, such as histone and DNA modifications, chromatin remodeling, and both proximal and distal transcriptional enhancers, contribute to regulation of heart development.

Due to the absolute necessity of this organ for survival, the knowledge of heart development and function has been the subject of intense investigation throughout decades of research. Several crucial factors involved in heart morphogenesis and disease have been identified. However, within the context of congenital heart disease (CHD) which occurs in nearly 1% of newborns, the current knowledge is still insufficient to diagnose and treat a majority of heart-related conditions [3]. One of the main reasons for this includes the lack of a comprehensive knowledge of factors involved in heart morphogenesis and the nature of their interconnectivity. 

The last two decades has seen an extraordinary progress in studying complex biological systems led by the genomics revolution. Of particular importance is the next generation sequencing (NGS) technology which allows the study of molecular interactions at a systems level and, more recently, at a single-cell resolution. The field of heart biology has no doubt benefited from this revolution as well, with which rapid progress has been made in the understanding of mechanisms of heart development, regeneration, and disease.

Here we review the recent contributions of NGS-based genomics studies into unraveling key mechanisms in heart development, regeneration, and disease, starting from early cardiac cell differentiation towards adult heart maturation and regeneration, based on evidence from embryonic stem cells (ESC) in vitro as well as various model organisms including zebrafish, mouse, chicken, and human. We further discuss the application of NGS techniques in clinical research that allowed the identification of novel genetic and epigenetic determinants of CHD. 

## 2. Applications of Next Generation Sequencing to Study Heart Development

### 2.1. Cardiac Cell Specification and Differentiation

The generation of mouse (mESCs) and human (hESCs) ESCs have provided invaluable tool to study developmental processes and pathological development of early mouse and human embryos. ESCs recapitulate pre- and post-gastrulation cardiogenic events in vitro up to the formation of mature CMs, overcoming the difficulty of tracking developing cardiac cells in vivo. Of note, comparative transcriptomic studies between CMs derived from mouse and human revealed that mES-CM and hES-CM cultured for 20 days correspond to E14.5 mouse hearts, whereas hES-CM cultured for nearly one year shows a similar gene expression profile as E18.5 CMs [4]. Moreover, single cell profiling revealed that the cardiac progenitor cell population consists of a heterogeneous population of cells at different states of differentiation at a given time [5]. These observations thus revealed the caveat of in vitro systems, in which molecular profiles can be biased depending on culture conditions and the origins of the cells. In order to overcome these discrepancies, it is crucial to relate the observations to that of in vivo systems. To provide a more complete overview of in vivo heart development, the generation of multiple transgenic lines in model organisms, such as zebrafish and mouse, has allowed the isolation of specific lineages of cardiac cells at various stages of heart development.

**Figure 1:**
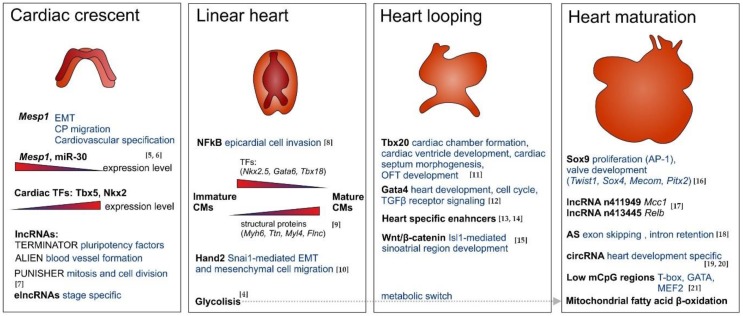
Genome-wide studies at different stages of heart development. Next Generation sequencing (NGS) studies at different stages of heart development facilitate the discovery of transcriptional regulators including transcription factors (TFs), enhancers as well as long non coding RNA (lncRNA), enhancer-associated lncRNAs (elncRNA), microRNA (miRNA), and chromatin modifiers driving stage-specific processes of cardiac development from early cardiac progenitor cells (CPs) differentiation up to heart maturation. AS: alternative splicing; circRNA: circular RNA; CMs: cardiomyocytes; EMT: epithelial to mesenchymal transition [4,5,6,7,8,9,10,11,12,13,14,15,16,17,18,19,20,21].

In the developing embryo, cardiac precursor cells are found before gastrulation and are located in the lateral posterior epiblast. Gastrulation, which begins with the formation of the primitive streak (PS), leads to the formation of three germ layers: ectoderm, mesoderm, and endoderm. The heart field precursors (cardiac progenitors) migrate anteriorly and laterally from the PS to form the anterolateral plate mesoderm that undergoes rapid migration towards the midline. The population of cardiac progenitor cells (CP) organizes into a crescent, with a peak of the crescent located cranially. Subsequent fusion of the midline gives rise to the linear heart tube. Within the cardiac crescent, the first heart field (FHF) cells differentiate and proliferate during linear heart tube formation, contributing to the left ventricle and partially to the atria. In turn, cardiac progenitors of the second heart field (SHF) cells contribute to heart tube elongation and further development of the right ventricle, outflow tract (OFT), atria, and inflow myocardium (Figure 1) [2]. 

Mesoderm posterior basic helix-loop-helix (bHLH) transcription factor 1 (Mesp1) marks CP that in mice give rise to FHF around E6.5, whereas Mesp1-positive cells at E7.25 form SHF. To investigate transcriptome changes at the earliest stages of CP specification, single-cell RNA-seq (scRNA-seq) was performed from fluorescence-activated cell sorting (FACS)-sorted Mesp1 positive cells from mouse embryos at E6.5 and E7.25 both in wild-type and *Mesp1*-KO background [5]. Transcriptome analysis of *Mesp1*-KO cells revealed developmental block in the gene program of epiblast cells hallmarked by the upregulation of pluripotency markers including *Nanog*, *Eras*, and *Oct4* as well as markers of epiblast *Epcam*, E-*Cadherin*, *Cdh1*, and *Cldn6*. Conversely, *Snai1* and *Zeb2* involved in EMT and *Etv2*, *Hand1*, *Myl7*, *Gata4*, *Flk*, and *Pdgfra* responsible for cardiac commitment were downregulated as compared to wild-type, indicating that Mesp1 is required to exit the pluripotent state of epiblast and drive EMT, CP migration, and cardiovascular specification. Interestingly, scRNA-seq showed that Mesp1-positive CP are molecularly heterogeneous, suggesting the presence of distinct subpopulations committed to the different cardiovascular lineages [5]. In a related study, single-cell complementary DNA (cDNA) sequencing in mouse embryo at E7.5 and E8.5 stages of development pinpointed the role of Mesp1 in early CP revealing that its expression progressively decreases during heart development, whereas expression of cardiac TFs including Tbx5 and Nkx2.5 increases [6]. Besides multiple TFs, the study also identified miRNAs including let-7, miR-140, miR-181, miR-30, miR-205, miR-103, and miR-22 which are conserved across multiple species from fly to human. In support of this, another RNA-seq study of chicken embryonic heart at six stages, starting from cell differentiation to heart maturation, showed that miR-30 was more highly expressed at earlier developmental stages, suggesting its potential function at early cardiogenesis events [6]. In another study, RNA-seq performed on differentiating human pluripotent cells to early cardiovascular progenitors identified 406 novel potential noncoding RNAs (ncRNAs) [7]. Further analysis revealed 75 stage-specific long noncoding RNAs (lncRNAs) with partial sequence conservation across multiple species including TERMINATOR, ALIEN, and PUNISHER, specific for pluripotent stem cells, vascular progenitors, and endothelial cells. Loss-of-function experiments in mouse embryos and human cells identified that TERMINATOR blockage resulted in downregulation of pluripotency factors (*Oct4*, *Sox2*, *Nanog*). On the other hand, ALIEN knock-down resulted in upregulation of extracellular matrix (ECM) genes and downregulation of genes related to blood vessel development, whereas PUNISHER knockdown affected mitosis and cell division genes and increased ECM encoding genes [7]. 

Another approach to study the mechanism of early cardiac progenitors specification was performed in mouse and human ESC differentiated to cardiac mesoderm, cardiac precursors, and finally CMs. These studies combine scRNA-seq/RNA-seq, assay for transposase-accessible chromatin with sequencing (ATAC-seq), and chromatin immunoprecipitation followed by high throughput sequencing (ChIP-seq) on multiple modified histones to allow simultaneous assessment of transcriptome and chromatin dynamics during early stages of heart development [7,22,23]. Such an approach has the power to identify novel candidate factors along with their interaction networks. As a notable example, RNA-seq was performed on differentiating mouse embryonic stem cells and adult hearts. Besides the identification of novel transcripts, the overlap of RNA-seq data with p300 ChIP-seq data allowed the identification of enhancers that are activated during cardiac differentiation and whose activity correlated with the expression changes of enhancer lncRNAs (elncRNAs). Knock-down of two selected enhancers in neonatal cardiac fibroblasts resulted in decreased expression of their putative targets including myocardin and *Smad7* [24]. A similar study in mESC [23] combined RNA-seq and ChIP-seq on epigenetic marks H3K4me3 (active promoters), H3K4me1 (promoters and enhancers), H3K27me3 (inactive promoter), and H3K27ac (active enhancers) at different stages of cell differentiation. Transcriptomic profiles of polyadenylated genes at each stage and their Gene Ontology (GO) term enrichment was, as expected, specific to the corresponding stages of CM differentiation. A number of known and novel miRNAs and lncRNAs were identified at specific stages of differentiation. Interestingly, the expressions of these lncRNAs were correlated with the expression of neighboring genes (including *Hand2*, *Gata6*, *Myocd*), pointing to the role of cis regulation during differentiation of heart progenitors. On the other hand, the ChIP-seq analysis revealed dynamic changes associated with promoter and enhancer activities. Combined analyses between gene expression and epigenetic profiles showed that chromatin patterns could distinguish functionally distinct genes with a similar expression pattern. Classification of enhancers as active (H3K27ac+, H3Kme1^+/−^) and poised (H3Kme1+ only) showed that only a small part of identified stage-specific enhancers was active. To test whether the activity of enhancers correlates with stage-specific gene expression, the single nearest gene was assigned to each of them. As expected, these enhancer-associated genes were enriched in GO terms specific to progressive cardiac differentiation stages. A portion of these cell-specific enhancers were dynamically changed during differentiation stages, suggesting that they may play a role in CM specification. In another study, ATAC-seq [25] and Hi-C [26] was incorporated to provide the information on chromatin accessibility and chromatin architecture. In combination with RNA-seq, the authors studied the process of differentiation of hESCs into CMs. Their analyses showed that in differentiating hESCs, heterochromatin regions pack more tightly within cis chromosome territories (intra-chromosomal contacts), whereas inter-chromosomal interactions (trans) are likely to occur between active regions localized in the proximity of cardiac-specific genes, such as titin (*TTN*). In general, collective results from RNA-seq, ATAC-seq, and Hi-C revealed that changes in chromatin accessibility occurred concomitantly with changes in RNA expression and large scale genome organization [27]. 

In summary, the combinatorial analyses between gene expression profile and epigenomics and/or chromatin architecture has not only identified novel factors implicated in CM specification and differentiation, but also revealed the dynamics in chromatin landscape associated with the progression in the acquisition of CM identity. 

### 2.2. Insights into Heart Morphogenesis

Following specification and differentiation, CMs migrate to the midline, forming the linear heart tube. Extension and rightward looping of the linear heart tube allows cranial positioning of the atria with respect to the ventricles (early morphogenesis). Further remodeling events modulate chamber formation, septation, and valve development, resulting in the formation of the four-chambered heart (late morphogenesis). NGS-based studies have contributed valuable insights into these two processes, revealing dynamic changes in the transcriptome and epigenome profiles in different cell lineages of the heart. 

#### 2.2.1. Early Morphogenesis

Single-cell RNA-seq of αMHC-GFP murine E9.5, E12.5, and postnatal (P1) hearts revealed that early stage heart cells are enriched in genes encoding for cell cycle, cardiac cell differentiation, and cellular migration. On the other hand, genes upregulated in P1 are linked to metabolism and structural proteins. Moreover, a wide spectrum of cell cycle markers is expressed at early stages followed by continuous decrease in their expression, supporting the existence of heterogeneous population of CMs at early stages of development [5] and their transition into a mature form. The study also revealed that CMs exhibiting high cell cycle activity had low expression of structural proteins (*Myh6*, *Ttn*, *Myl4*, *Flnc*) and high expression of cardiac embryonic TFs (Nkx2-5, Gata6, Tbx18), whereas cells with low cell cycle activity showed the reverse expression profiles [9]. To better understand the role of SHF transcriptional regulators involved in atrioventricular (AV) septations, ChIP-seq binding maps of Tbx5 and Gli1 were intersected from mouse hearts isolated at E9.5. This data revealed a cis-regulatory region of *Foxf1a* bound by both Tbx5 and a Gli1, a Hedgehog transcriptional regulator, suggesting the crosstalk between Foxf and Hedgehog signaling in AV septation [28]. Single-cell transcriptomic studies performed in mouse heart at E8.5, 9.5, and 10.5 allowed the identification of the origin of single cardiac cells that have been partitioned in 17 subpopulations based on cell type and anatomical location [29]. A typical transcriptional signature has been established for CMs (*Tnni3*^+^, *Nebl*^+^, *Tnnc1*^+^, *Cryab*^+^, *Myl4*^+^), EC (*Emcn*^+^, *Cdh5*^+^, *Icam2*^+^, *Plxnd1*^+^, *Ecscr*^+^), EP (*Aldh1a2*^+^, *Upk3b*^+^, *Upk1b*^+^, *Kcne1l*^+^, *Tmem255a*^+^), and mesenchymal cells (*Cthrc1*^+^, *Pdgfra*^+^, *Postn*^+^, *Sox9*^+^, *Fbln2*^+^).

Another elegant study performed in the zebrafish heart applied the TOMO-seq method which is based on deep RNA sequencing of individual cryosections [30]. This technique provides additional spatial information, allowing the assignment of location to the transcriptome profile. Applying this technique to study the zebrafish heart at 48 hours post-fertilization (hpf), Burkhard and colleagues [15] identified differentially expressed genes between different cardiac sub-compartments including ventricle, atrium, atrioventricular canal (AVC), and sinoatrial regions. GO enrichment analysis showed that processes related to oxygen transport and tricarboxylic acid cycle genes are enriched in the ventricle, suggesting the conversion to aerobic oxidative phosphorylation that occurs during cardiac looping. On the other hand, the sinoatrial region was enriched in genes involved in proepicardium development, hippo signaling, and Wnt-signaling. Functional studies in transgenic lines and *Isl1* mutants revealed that Wnt/β-catenin signaling is locally activated in sinoatrial region and depends on Isl1 activity [15]. Genome-wide studies of *Hand2*, including ChIP-seq of *Hand2* and RNA-seq in wild-type and *Hand2*-KO embryos at E10.5, revealed that this TF—which is expressed in the myocardial compartment of the right ventricle, OFT, the epicardium and valve progenitors—targets genes involved in AVC cardiac cushion development. The effect of Hand2 was found to be mediated by *Snai1*, an EMT and mesenchymal cell migration regulator, which was found to be under transcriptional regulation of *Hand2* [10]. A very general overview of genes expressed at different stages of zebrafish heart development including heart looping (~30 hpf), CM maturation, initial trabeculation, AVC canal formation (30–55 hpf), valve formation (~55 hpf), and heart maturation (~72 hpf) identified stage-specific gene expression patterns that were organized into regulatory gene networks driving heart development [31]. Based on this approach, a number of TF motifs—including those bound by Foxa, Sox, Klf, and Tbx—were identified nearby associated genes pointing its potential importance in driving heart development.

#### 2.2.2. Late Morphogenesis

The onset of transcriptional program has been found to control the process of heart maturation comprising remodeling and maturation of the chambers, septation, and finalization of valve formation. 

At E11.5 stages of mouse embryo development when atrial septation, muscular interventricular septum formation and early outflow septation occurs [32]. Genome-wide Tbx20 binding maps in the endocardium, myocardium, and cushion mesenchyme were enriched near genes involved in cardiac chamber differentiation, cardiac ventricle development, and cardiac septum morphogenesis, including those involved in EMT and OFT development [32]. The overlap of Tbx20 binding sites with ATAC-seq data identified open chromatin regions, while transcriptomics data identified putative promoters and enhancer elements associated with direct Tbx20 binding to the accessible chromatin regions. The intersection of orthologous conserved regions from Hi-C performed on human fibroblast with heart-specific Tbx20 peaks identified enhancers driving the expression of Vcan, a regulator of cell migration and invasion in cardiac septation [11]. To study coronary vessel development at embryonic stage E11.5, mouse immortalized epicardial cell lines were generated from wild-type and Tgfβr3^−/−^ mice. RNA-seq analysis of control and cells stimulated with ligands inducing TGFβR3-dependent invasion revealed NF-κB signaling pathway as a main player in epicardial cell invasion [8].

Single-cell transcriptomics of cells isolated from mouse hearts were performed at several embryonic days E9.5, E11.5, E14.5, and E18.5 corresponding to heart development stages starting from heart looping until septation and metabolic remodeling [4]. This profiling identified stage-specific cell populations and their gene signature. Highly enriched genes in CMs included sarcomere protein genes and *Sh3bgr*, whereas in endothelial cells (ECs) *Klhl4*, *Gpr116*, *Pecam1*, and *Cdh5* revealed the highest expression as compared to other cells types. ECM proteins were enriched in fibroblasts, which appear in the heart by E12.5. The analysis of transcriptional profile of ventricular CMs, revealed the presence of two subpopulations, one with a strong proliferative capacity and represented the most numerous population between E9.5 and E14.5, and another ECM-enriched CM subpopulation which increased in proportion during heart development. Moreover, the differences were observed in transcriptional profiles of CMs isolated from either ventricle or atrium and their dynamics during heart development. In atrial CMs, genes related to adult CM physiology—such as *Gja1* and *Csq2*—progressively increased during development, whereas the expression of *Wnt2*, a regulatory of early atrial development, decreased. In ventricular CMs, a clear switch from embryonic glycolysis to mitochondrial fatty acid β-oxidation was observed from E9.5 to postnatal stage, hallmarked by an increase of *Fabp3*, *Fabp4*, and *Cox8a*. Similarly, ventricular CMs revealed heterogeneity in terms of distinct patterns of genes expression over development. A cluster of genes related to heart maturation and fatty acid oxidation revealed increased expression from E9.5 until postnatal stages, while another cluster enriched in glycolysis and cell proliferation showed converse expression pattern. A third gene cluster enriched in mitochondrial function and calcium metabolism showed higher expression during late heart maturation and postnatal stages. Differences were observed between genes expressed in cells from left versus right ventricle, the latter being enriched in genes related to ECM organization. Interestingly, transcriptomic studies in Nkx2.5^+/−^ mouse embryos showed that haploinsufficiency of this TF associated with CHD delays maturation of CMs and ECs as compared to wild-type individuals [4].

Besides genes, regulatory elements play an equally important role in driving heart morphogenesis. To investigate the role of enhancers during cardiac looping, genome-wide maps of p300 binding from E11.5 mouse embryo hearts were identified. As a comparison, p300 ChIP-seq was also performed in forebrain, midbrain, and limb tissues. Surprisingly, multi-vertebrate genome alignment revealed that only 6% of candidate heart enhancers overlap strongly conserved interspecies genomic regions, whereas in other tissues such as forebrain, midbrain, and limb, the conservation is much stronger reaching 44%, 39%, and 30%, respectively, suggesting that cardiac enhancers exhibit relatively weak evolutionary conservation. Interestingly, the study shows that 84% of putative heart enhancers identified by p300-ChIP-seq were active in the developing hearts of transgenic enhancer assay mice. However, when extreme evolutionary approach was used to predict enhancer regions, only 3% of them were confirmed in in vivo enhancer assay. Interestingly, most of the biologically-validated enhancers were localized near genes involved in heart development [13]. Complementary to studies in mice, ChIP-seq of p300 in human fetal heart at 16 weeks and adult heart enabled the identification of 2233 human heart enhancer candidates, from which 1082 were specifically present in fetal hearts. 21% of fetal human enhancer candidates had their orthologous sites localized within significant peaks in mouse genome. From 65 enhancers tested in transgenic mice at stage E11.5, 43% showed heart-specific expression, thus identifying human heart-specific enhancers at an early stage of morphogenesis [14].

ChIP-seq study was performed on E12.5 heart ventricles to identify the downstream targets of Gata4 transcription factor which is known for its role in cardiac morphogenesis and CMs proliferation [12]. Gata4 binding sites were found to strongly overlap previously published p300 binding profiles, suggesting that Gata4 regions contain functional transcriptional enhancers. RNA-seq performed in *Gata4*-KO hearts revealed a number of genes downregulated compared to wild-type. However, only less than one-third of these genes were associated with enhancers occupied by Gata4. This group of genes was enriched for heart and muscle development GO terms. Twelve candidate regions were tested in transient in vivo transgenic enhancer assay, out of which seven were active in the heart. Moreover, transient transgenic assay of enhancers with mutated Gata4 motifs suggested that the Gata4 motifs within validated cardiac enhancers seemed to be crucial for their activity. Interestingly, the intersection between Gata4 ChIP-seq and H3K27 ChIP-seq in wild-type and *Gata4*-KO showed that Gata4 promotes deposition of H3K27 at distal Gata4 enhancers. Gata4 ChIP-seq on embryo and adult hearts revealed the presence of stage-specific Gata4 peaks. Fetal-specific peaks were associated with heart development, positive regulation of cell cycle, and TGFβ receptor signaling [33]. In another study involving multiple TFs including Gata4, Mef2a, Nkx2.5, Tbx5, and Sfr, ChIP-seq was performed in mouse HL1 cell line mimicking maturated CM [13]. Comparisons between the binding profiles of these TFs revealed that a number of genomic regions are co-occupied by more than one TF, indicating regulatory “hot-spots”. Functional enrichment analysis of genes associated with these hot-spots revealed GO terms related to heart function and development, supporting the role of hot-spot regulatory regions in heart morphogenesis. Several of these regions exhibited both heart-specific and non-specific enhancer activities in transient transgenic assays in vivo, in E10.5 mouse embryos. The enhancers were further classified into three groups—occupied only by multiple TFs, occupied by multiple TFs and p300, and occupied by p300 only. Interestingly, a major part of enhancers tested in vivo were not occupied by p300, suggesting the existence of p300-independent cardiac enhancers [34]. In a similar approach, RNA-seq and ChIP-seq of Sox9, a cardiac TF expressed in the newly formed mesenchyme of the developing heart valves, revealed that Sox9 controls proliferation-related genes including AP-1 complex as well as a number of TFs involved in valve development including Twist1, Sox4, Mecom, and Pitx2 [16]. 

Integrative analysis of RNA-seq, ChIP-seq (H3K27ac, H3K9ac, H3K4me3, and H3K36ac), 5-hydroxymethylcytosine sequencing (5hmC-seq) and whole-genome bisulfite sequencing (WGBS) data from fetal, infant and adult human heart samples, characterized CM-specific putative enhancers adjacent to genes related to cardiac muscle contraction and morphogenesis [12]. Dynamic genic CpG methylation (mCpG) was observed in around 10% of unmethylated CpG between prenatal and postnatal hearts. Methylome changes negatively correlated with expression levels of genes involved in CM maturation. Low mCpG regions in CM were enriched in motifs of cardiac TF, such as T-box, GATA, and MEF2. Large overlap has been found between cis-regulatory regions identified as low mCpG regions and enhancers predicted by chromatin marks. Interestingly, those genomic regions were localized in the proximity of genome-wide association studies (GWAS)-identified cardiac arrhythmia and CHD genes including *HCN4*, *KCNE1*, *KCNH2*, *KCNJ2*, *KCNQ1*, and *SCN5A* [21].

The role of ncRNAs has been extensively studied in maturing hearts. RNA-seq of total RNA from different mouse organs (heart, liver, skin) identified 117 heart-enriched lncRNAs [33]. Moreover, a comparison of adult heart and E14 hearts revealed 157 differentially expressed lncRNAs whose expression were correlated with changes in the expression of mRNA encoding transcripts localized within 10 kb. This analysis established lncRNA-mRNA pairs whose function was related to cardiac development and metabolic pathways as well as TFs, such as NFKB and CREB1. Functional validation in C2C12 mouse skeletal fibroblast line confirmed that knockout of two of them, lncRNA n411949 and lncRNA n413445, leads to an increase in the expression of their cis-regulated targets *Mcc1* and *Relb*, respectively [17]. Besides mRNAs, other species of transcripts have recently surfaced as important regulators of biological processes. One such type of noncoding RNAs are the circular RNA (circRNA) whose suggested functions include suppression of miRNA activity and scaffolding for RNA-binding proteins. RNA-seq data from adult and fetal tissues allowed the identification of a number of tissue-specific circRNA in adult as well as in fetal human tissues. These include a number of cardiac-specific and differentially expressed putative circRNA [19]. RNA-seq analyses on human, mouse, and 28-day differentiation time-course hESCs-derived CMs generated a detailed circRNA expression landscape in the heart [20]. Interestingly, many of these circRNAs were found to be cardiac-specific and their abundance correlated with that of their cognate linear RNAs which corresponded to several key cardiac genes. The identification of this class of RNAs opens new areas of research into their role in regulating heart development and function. 

A transcriptome-wide comparison of human fetal and adult heart samples identified specific changes in alternative splicing (AS) events. The analysis showed that intron retention and exon skipping were more common in fetal hearts compared to adult. Specific AS were identified for fetal and adult hearts, hallmarking more frequent events in protein-coding genes than lncRNAs. Fetal-specific alternatively spliced genes were enriched in biological processes related to cell cycle and chromosome organization, whereas those enriched in adult hearts were related to protein folding, translation, and ribonucleoprotein biogenesis [18]. 

### 2.3. Lessons from Next Generation Sequencing Application in Clinical Studies of Congenital Heart Disease 

The development of next generation sequencing techniques has emerged as a powerful tool to study the sequences of either entire exomes by whole exome sequencing (WES) or selected disease-specific genes using targeted exon sequencing. These two methodologies have revolutionized our understanding of inherited Mendelian diseases, providing a detailed picture of genomic variations as compared to SNP-limited sequencing in GWAS. Non-congenital forms of cardiac disorders which are beyond the scope of this review have been covered in several excellent articles which readers are directed to [35]. WES and targeted exon sequencing studies in CHD patients allowed to identify a number of exonic mutations in genes previously associated with heart development and its malformations validating GWAS and functional studies in model organisms. Of note, in a major part of the studies, sequencing of exons was often limited to genes previously implicated in heart development and cardiac malformations, likely due to the presence of no significant differences between de novo mutations in control and CHD patients. Importantly, in several studies, WES applied in large cohort of patients or familial forms of CHD enabled to characterize novel mutations associated with cardiac developmental anomalies [36]. 

WES and targeted exon resequencing have been applied in studying multiple CHD phenotypes, including atrial septal defects (ASD), bicuspid aortic valve (BAV), patent ductus arteriosus (PDA), tetralogy of Fallot (TOF), left-ventricular outflow tract obstructions (LVOTO), and left ventricular non-compaction (LVNC). In most cases, novel mutations were found within genes already associated with CHD (Table 1). Nevertheless, several studies provided novel insights into the role of epigenetic factors and ncRNA in CHD. Targeted exon resequencing of patients with BAV identified 97 candidate genes including 33 disease-causing mutations within *GATA4/5*, *APC*, *JAG1*, *NOTCH1/2/3*, *PAX8*, *SOX9*, *TBX5*, and *WNT4* out of each 26 genes were not previously associated with BAV [37]. Another study in 32 BAV patients revealed novel G4297A variant of *NOTCH1* and putative pathogenic *ADMTS5* C935A variant [38]. Targeted sequencing of 16 known strictly CHD-related genes in 68 CHD patients identified variants previously linked to CHD including *NKX2.5*, *ZFPM2*, and *MY6* as well as novel mutations in *GATA4*, *NKX2.5*, *NOTCH1*, and *TBX1* [39]. Targeted resequencing of 181 individuals from 41 families with LVOTO revealed disease associated and co-segregating variants of *NOTCH1*, *ARHGAP31*, *MAML1*, *SMARCA4*, *JARID2*, and *JAG1* in 12 families [40]. In a WES study performed on 2871 CHD cases including parent-offspring trios, a number of rare inherited mutations were identified in 1789 cases [41]. Of these, mutations in seven genes surpassed the threshold for genome-wide significance (*CHD7*, *KMT2D*, *PTPN11*, *FLT4*, *NOTCH1*, *RBFOX2*, *SMAD6*), and mutations in another 12 genes not previously implicated in CHD had more than 70% probability of being disease related (*GATA6*, *ELN*, *CCDC154*, *SLOCO1B3*, *GPBAR1*, *PTEN*, *RPL5*, *NSD1*, *SAMD11*, *C210RF2*, *NODAL*, *SMAD2*, *H1F00*, *FRYL*, *KDM5B*, *POGZ*, *SOS1*, *TBX18*). Targeted resequencing of 22 CHD patients also allowed the identification of 65 mutations, out of which 13 were not previously reported [42]. Interestingly, the development of custom clinical-grade NGS assay for CHD mutation identified 88 genes and 40 miRNAs were used in the array [43]. An elegant integrative study, in which targeted resequencing was performed for genes selected based on RNA-seq data obtained from mouse heart at E14.15, revealed that human orthologs of highly expressed mouse cardiac genes related to H3K4 production, removal and methylation including *WDR5*, *MLL2*, *KDM5A*, *KDM5B*, *SMAD2*, *UBE2B*, and *RNF20* were affected by mutations in CHD patients [44]. Interestingly, novel genes have been associated with hypoplastic left heart syndrome (HLHS) by combining mouse forward genetics and WES [45]. These findings, confirmed further in functional studies in zebrafish and mouse, showed that Sap10 mediates left ventricular hypoplasia, whereas Pcdha9 increases the penetrance of aortic valve malformations. 

## 3. Next Generation Sequencing in Heart Regeneration

### 3.1. Heart Regeneration—Decades of Intensive Studies

The mammalian heart has been considered to be incapable of any regenerative response due to the withdrawal of the adult mammalian cardiac cells from the cell cycle. However, recent studies disproved that longstanding dogma and clearly showed that mammalian heart is not a post-mitotic organ. Neonatal murine heart can efficiently regenerate a fully functional myocardium [56,57], while the adult human heart is capable of modest self-renewal [58,59,60]. Nevertheless, the self-renewal of the human heart is unable to replenish damaged tissue, as it is still one of the least regenerative organs in the body [61]. During tissue remodeling following ischemia-induced myocardial infarction, most of the lost CMs is replaced with collagen-rich scar tissue that stabilizes the wound area but simultaneously weakens cardiac output due to lack of contractility [62]. The limited capacity of the human heart to activate endogenous regeneration processes is the main cause of mortality and morbidity throughout the world, thus making heart failure a serious public health problem [63,64].

In contrast to humans, lower vertebrate species like the zebrafish exhibit remarkable heart regeneration upon adulthood. During the last 15 years, the zebrafish has become a clinically relevant model organism in the study of heart regeneration. Its heart recovers efficiently from different types of induced insult including ventricular resection [65,66], genetic ablation of CMs [67], and ventricular cryoinjury [68,69,70]. The cardiac wound resulting from cryoinjury initially forms a fibrin clot, which is gradually replaced by de novo regenerated myocardium [68,69]. Lineage-tracing studies have clearly proved that new cardiac tissue in zebrafish is generated from differentiated CMs [71,72]. The same result has been observed during cardiac regeneration after genetic ablation of CMs [67] and during the regeneration of neonatal mouse heart [57].

**Figure 2:**
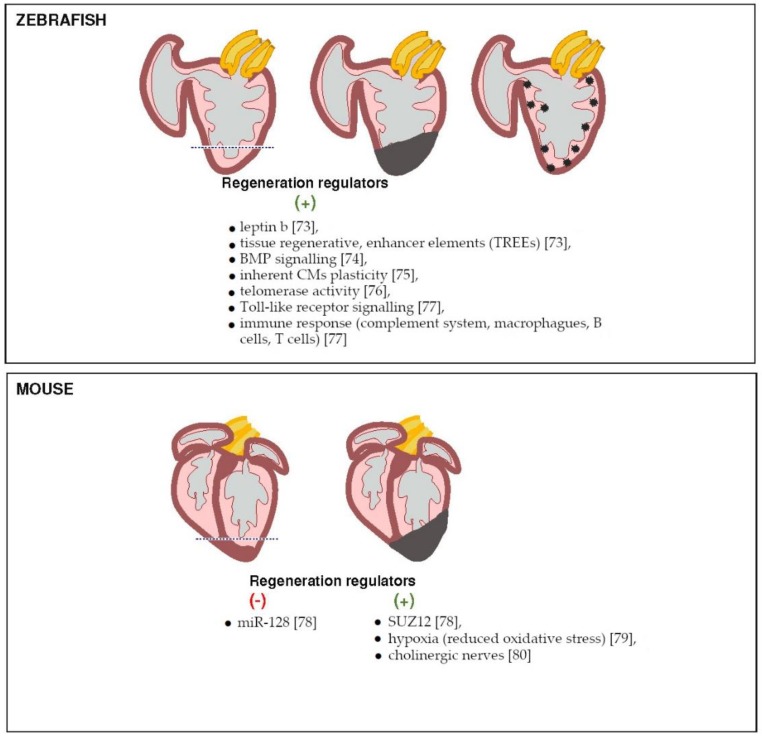
Insights into heart regeneration gained from NGS-based studies. Different approaches for cardiac injury induction in adult zebrafish (upper) and neonatal and adult mouse used to investigate heart regeneration. NGS studies on cardiac repair in lower vertebrates (zebrafish) and mammals (mouse) have identified molecular regulators including driving genes, enhancers, miRNA, as well as cellular and environmental factors directing efficient heart regeneration [73,74,75,76,77,78,79,80].

Given the identification of existing CMs as the dominant source of the regenerated cardiac tissue in the adult zebrafish heart, intensive studies have been conducted within the field in order to identify the molecular determinants involved in CM proliferation. The employment of genomics approaches in several landmark studies in the field of heart regeneration has generated unprecedented key insights into molecular mechanisms underlying the regenerative capacity of the heart. Profound analysis of the molecular responses at the genomic, epigenetic, and transcriptional level following induced cardiac injury in established model organisms has become a potent tool in uncovering molecular bases of heart regeneration. The knowledge gained from the application of next generation sequencing technology has allowed the identification of candidate drivers of heart repair, which may stand as potential targets for triggering or boosting CMs renewal in adult mammals, including humans (Figure 2).

### 3.2. Molecular Mechanism of Cardiac Regeneration

Heart regeneration is a highly complex process involving the interactions of multiple cellular and molecular pathways which activate the proliferation of pre-existing CMs to replenish cells lost during cardiac injury [57,71,72].

Taking advantage of previous knowledge on the role of pre-existing CMs as the dominant source of regenerating heart muscle tissue, NGS techniques have been successfully employed in the identification of changes occurring at the genome-wide level in the heart upon its response to induced organ injury. This approach has yielded a better understanding of the mechanisms regulating efficient cardiac repair. The heart of the neonatal mice is capable of effective remodeling and complete morphological and functional regeneration until postnatal day 7 [57,81]. To identify a gene expression signature of the regenerating neonatal heart, Haubner and colleagues performed RNA-seq on untouched, sham-operated, and injured P1 murine hearts [81]. Comparison of transcriptome profiles between sham-operated and injured neonatal hearts revealed only a small number of differentially expressed genes (294 genes). Nevertheless, most of the genes differentially expressed in left anterior descending artery (LAD)-ligated mice have been annotated to the cell cycle progression, mitosis, and DNA replication, indicating that injured murine heart carries a gene expression signature corresponding mostly to the activated cell cycle machinery which is responsible for the prompt and efficient organ regeneration. In contrast to mouse, the zebrafish retain their tremendous regenerative potential throughout life [65,66,67,68,69,70,71,72]. In order to determine genes that are induced during tissue regeneration, Kang and colleagues performed RNA-seq analyses on uninjured and regenerating adult zebrafish hearts and fins [73]. This allowed the identification of potential driver genes of tissue repair. They discovered that leptin b (*lepb*) was upregulated upon local ventricle injury in the endocardium of regenerating heart. Interestingly, ChIP-seq against H3K27ac (marker of active enhancers) in regenerating heart and fin tissues identified a short DNA element located 7 kb upstream of the *lepb* coding sequence which was shown to play an important role in directing gene expression upon tissue regeneration. Moreover, genome-wide H3K27ac survey enabled to recognize additional 1–2 kb intergenic regulatory elements named tissue regeneration enhancer elements (TREEs) which serves to activate and maintain their target genes upon injury and subsequently alleviate them when regeneration is accomplished.

The knowledge on potential driving factors of regeneration has been significantly enriched by the application of TOMO-seq [30]. Using this technique, Wu and colleagues identified two border zones in the regenerating zebrafish heart that indicate distinct expression profiles [74]. The transcriptome profile revealed the existence of two spatial sub-clusters of genes, one corresponding to the border zone further apically near the injury area (BZinj), and another in the border zone further basally near the uninjured myocardium (BZmyo). Subsequent analysis revealed that apical BZinj zone displays an expression profile characteristic for fibrosis and vasculogenesis, with the upregulation of genes abundantly expressed in vascular endothelium (*vwa1*, *cldn5b*, and *mmrn2b*) and smooth muscle markers (*myl6*, *junbb*, *crip2*, and *tagln*). On the other hand, embryonic myocardial genes (e.g., *nppb*, *vmhc*, *actc1*, and *desma*), as well as growth factors and cell cycle driving genes were induced in the BZmyo. It has been shown that bone morphogenetic protein (BMP) signaling is activated in the zebrafish heart in response to cardiac injury. BMP signaling is required for cardiac regeneration, where it drives de-differentiation and proliferation of CMs at the wound border (BZmyo). A few genes known to be components as well as targets of BMP signaling—such as *acvr1l*, *smad1*, *id1*, *id2b*, *junbb*, *sall1b*, and *rgma*—were enriched in the two identified border zones. These results show that heart regenerative factors, which were required for prompt and efficient reconstruction of the organ, were essentially developmental factors which were re-activated in response to cardiac insult.

The role of noncoding RNAs have also been recognized in the context of heart regeneration. RNA-seq analysis performed by Huang et al. on uninjured mouse ventricles on P1, P7, and P28 resulted in the identification of miR-128, as the driver molecule implicated in cell cycle shutdown of mature CMs [78]. Identified target genes of miR-128 have been shown to be involved in cell cycle regulation. Comparison of downregulated mRNAs in miR-128OE (overexpressing) CMs with that of control hearts identified 87 putative target genes of miR-128 which carried the predicted miR target sequence at their 3’UTR. GO analysis revealed that nearly 30% of these genes belong to the “cellular process” category. Subsequent analysis further revealed that miR-128 can affect multiple pathways including cell cycle, cell communication, and cellular component movement. One of the most promising target genes of miR-128 in the heart is Suz12, a component of the chromatin modifier complex PRC2 (polycomb repressive complex 2), known for its crucial role in organogenesis [82]. MiR-128 directly regulates CM proliferation via its interaction with Suz12. Subsequent investigations revealed that inhibition of miR-128 in LAD-ligated mice hearts promotes the proliferation of pre-existing CMs via SUZ12 and improves endogenous cardiac regeneration after induced MI [78].

The knowledge that CMs are the main source of regenerating heart tissue has been well established. However, there have been conflicting ideas as to the source of the regenerating CMs. Using RNA-seq technique, Sánchez-Iranzo et al. have shown that CMs plasticity might be a key feature of the effective and precise cardiac regeneration [75]. In response to induced insult, trabecular CMs contribute to rebuilding the cortical layer of damaged myocardium. Previous studies have proposed that cortical myocardium regenerates exclusively from the same layer [83]. However transcriptome analysis conducted by Sánchez-Iranzo et al. have revealed that *tbx5a*-derived CMs, expressed only in trabecular layer of the adult myocardium, adopt the expression profile of cortical CMs upon relocation events. During the re-specialization of *tbx5a*-derived CMs, significant upregulation of *xirp2a* and *lama5*, molecular markers of the cortical CMs, takes place with concomitant downregulation of *tbx5a* gene expression. As in the developing heart of mice, the trabecular layer of myocardium had a lower rate of proliferation, thus the shift from trabecular to cortical phenotype might allow CMs to re-enter the cell cycle and boost repair processes after heart injury [75].

Several studies have shown that aerobic-respiration-mediated oxidative damage of DNA plays a crucial role in cell cycle re-entry of pre-existing CMs in response to cardiac insult [84,85]. Successful cell proliferation requires healthy telomeres and intact DNA. Using RNA-seq, Bednarek et al. showed that the loss of the telomerase enzyme responsible for restoring the telomere drastically decreases CMs proliferation in damaged zebrafish myocardium [76]. Transcriptome analysis demonstrated that knock-out of telomerase gene in zebrafish (tert^−/−^) results in the impairment of multiple pathways associated with cell proliferation. The expression of cyclins, cdks, replication licensing factors and processivity factors engaged in cell proliferation was significantly downregulated in tert^−/−^ injured hearts compared to that in wild-type. Interestingly, the transcriptome analysis did not disclose any meaningful dissimilarity in the upregulation of inflammatory cell markers between tert^−/−^ null mutant and wt. Moreover, the expression of endocardial (aldh1a2, il11a) and epicardial (wt1b, tbx18, fibronectin) developmental genes inducing CM proliferation was unaffected in tert^−/−^ compared to wt. Nakada et al. corroborated that oxidative damage of DNA has a negative impact on efficient heart regeneration [79]. RNA-seq analysis of hypoxic and normoxic left ventricle of the injured murine heart demonstrated a number of dysregulated pathways. Hypoxic adult animals displayed a significant upregulation of multiple genes involved in mitotic cell cycle, proliferation, angiogenesis, and vascular morphogenesis. Profound changes in transcriptome profile of normoxic and hypoxic animals suggest that hypoxic conditions and reduced oxidative damage of DNA induces proliferation of pre-existing CMs, allowing for efficient regeneration of adult murine heart.

CMs that build the heart and participate in its repair do not function in an isolated environment. They are under the influence of numerous other cell types populating the organ transiently or permanently. Transcriptome profiling of the neonatal mouse heart after mechanical denervation conducted by Mahmoud et al., revealed patterns for disruption of inflammatory gene expression that were commonly activated during heart regeneration [80]. RNA-seq data analysis disclosed that the most pronounced difference in gene expression between resected and resected plus mechanically denervated hearts was a dampening in the expression of markers for inflammatory genes mobilized in response to cardiac resection. Significantly downregulated in the injured, denervated heart were genes implicated in the innate immune response and chemotaxis (*IL1b*, *Cxcl5*, and *Pf4*). Simultaneously expression of several immune genes engaged in the adaptive immune response and lymphocyte mediated immunity (*Nfkb2*, *Bcl6*, *Slc11a1*, and *Icam-1*) remained unchanged in response to vagotomy. These transcriptional changes have been shown to be conserved across species, as cholinergic nerve-mediated regulation of heart repair has also been observed in the adult zebrafish.

Immune response and tissue inflammation have also been reported to play a key role in an effective cardiac repair in zebrafish [86,87] and neonatal mice [88,89]. The type of immune response and type of cells engaged in the injury repair at the liaison site guides the extent of injury repair [90]. Medaka, a teleost fish, is incapable of efficient heart regeneration [91]. Using RNA-seq technique, Lai et al. demonstrated that early immune response plays a key role in modulating consecutive events required for heart regeneration [77]. Detailed comparative transcriptomic analyses following cardiac injury in medaka and zebrafish identified the molecular factors that underlie the pronounced difference in regenerative capacity of these two phylogenetically related species. Overall response to cardiac insult is comparable in these two species. However, even though the global changes in gene expression after cryoinjury are similar between zebrafish and medaka, it is less powerful and considerably delayed in the latter. More in-depth analysis of the differentially expressed genes over time between the injured medaka and zebrafish (sample level enrichment analysis, SLEA) demonstrated that the immune response in zebrafish showed stronger activation of genes involved in the complement system, macrophages, B cells, T cells, and phagocytosis, while medaka showed a stronger activation of genes related to neutrophils and monocytes. In addition zebrafish showed a stronger and prolonged activation of genes involved in cell proliferation and angiogenesis. Pathway analysis based on genes differentially upregulated or downregulated in zebrafish compared to medaka enabled to identify putative canonical pathways, including phagocytosis, NF-kB, PI3K/AKT, NFAT, and Toll-like receptor signaling and upstream regulators potentially responsible for the differences in cardiac regenerative capacity.

Collectively, the use of genomic approaches based on NGS technologies yielded significant advances in our understanding of molecular mechanisms underpinning heart regeneration through the identification of novel genes and regulatory elements involved in injury and repair, as well as the elucidation of interactions between distinct cell types contributing to the regeneration process. Importantly, the extensive application of NGS methodology to both developmental and regenerative processes enables an exploration of the extent to which formation and rebuilding of the organ are controlled by the same genetic factors.

## 4. Conclusions and Future Perspectives for the Application of Emerging Next Generation Sequencing -Based Assays on Heart Biology

**Figure 3:**
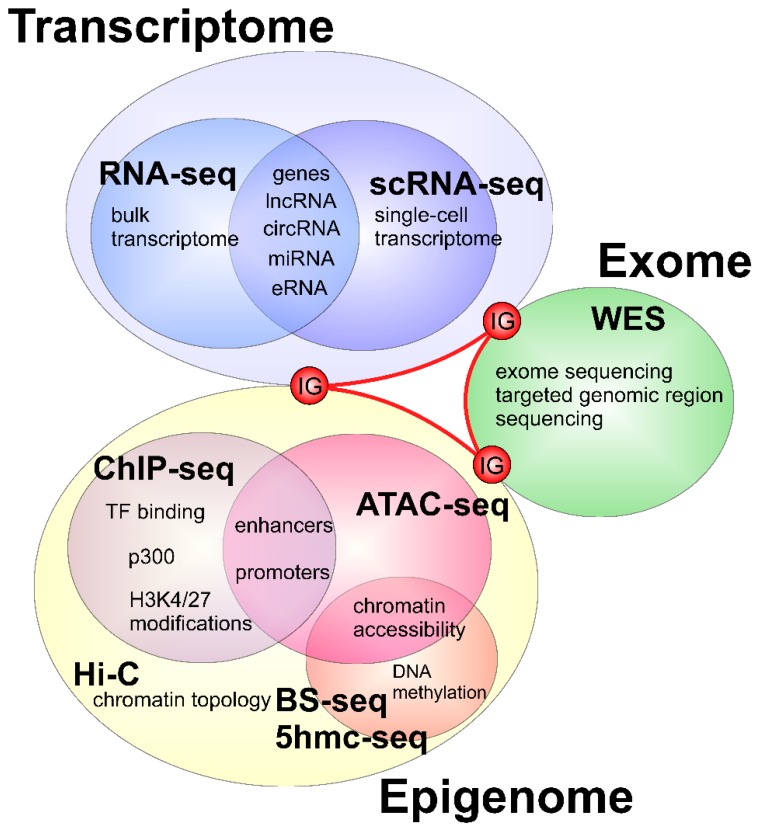
Perspective integrative genomic studies that may contribute to our understanding of molecular mechanisms of heart development and regeneration. The integration of transcriptomic (RNA-seq, scRNA-seq) and epigenetic (chromatin immunoprecipitation followed by high throughput sequencing (ChIP-seq), assay for transposase-accessible chromatin with sequencing (ATAC-seq), HiC, bisulfite sequencing (BS-seq), 5-hydroxymethylcytosine sequencing (5hmc-seq)) studies from model organisms with clinical data from congenital heart disease (CHD) patients (Whole exome sequencing (WES) or targeted genome sequencing) can provide novel insights into our understanding of molecular mechanisms driving heart development and the identification of genetic factors responsible for cardiac developmental anomalies. Integrative Genomics (IG).

The advent of genomics has brought about the development of next generation sequencing technology which has rapidly driven biological discoveries in many areas of research. Within the field of heart biology, NGS has expedited the discovery and annotation of novel genetic factors involved in the development and function of the organ. In addition, application of NGS in the clinical side has identified a number of genetic factors associated with heart conditions. Integrative and comparative multi-species studies, combining NGS approaches to investigate transcriptomic, epigenetic, and genetic determinants of physiological heart processes and their anomalies both in clinics and in model organisms should be addressed to further broaden our knowledge (Figure 3). The task which remains at hand is to perform functional characterization of these factors and study the mechanism by which they contribute to development as well as pathogenesis. Such effort will be particularly important to link disease phenotypes to their causative factor which is necessary in developing diagnosis methods and clinical therapies.

In recent years, the development of single cell analyses has increased the resolution with which cellular identity and function can be studied. Coupled with the NGS technology, single cell genomics has now propelled the discovery of novel insights into the diversity of cellular identity and states in tissues and organs, including the heart. Several recent key publications in this field have significantly advanced the understanding of various aspects of heart biology. In one of the first such applications, DeLaughter and colleagues [8] performed sequencing of single cells isolated from the murine heart at multiple developmental stages spanning the primordial heart tube stage to mature heart. Their analyses defined the various types of cardiac cell lineages present at various time points during heart development and identified molecular markers expressed at specific stages and in different regions of the heart. Furthermore, their study showed that the single cell analysis can also be applied to characterize the defects in CM maturation process in a defective heart as a result of Nkx2.5 mutation, suggesting potential application in medical diagnosis. In the field of heart regeneration, single cell genomics have been applied to profile specific subtypes of the cardiac cell lineage, the epicardium, which is known to play a role in heart regeneration [92]. Through this analysis, the essential role of Caveolin 1 in heart regeneration was established. In a more recent study, the application of single cell sequencing on very early cardiac specification events has helped to define the molecular process of lineage segregation in the various cardiovascular cell types in the mouse heart [5]. The CM has been the focus of most studies in heart biology, while considerably less is known about non-CM lineages. An interesting single cell study by Skelly and colleagues [93] looked at this population of cells and defined a number of distinct cell types based on their unique transcriptome profiles. The analyses revealed the heterogeneity of cellular sub-types within the major cardiac cell lineages, and by applying the information from ligand-receptor pairs dataset, established the extensive intercellular communication network between the different populations of heart cell types.

While there are many other single-cell RNA-seq studies which are not mentioned in this review, these examples show the power of this technology to drive future discoveries in the field of heart biology. Coupled with the advances of CRISPR-based barcoding techniques which are increasingly used to trace the developmental progression of various cell lineages [94,95], a comprehensive view of heart development, regeneration, and disease mechanisms might be closer than we think. 

## Figures and Tables

**Table 1:** Whole exome sequencing (WES)-identified genes affected in patients with diagnosed congenital heart disease (CHD).

Heart Malformation	Identified Disease Genes	Reference
362 parent-offspring trios with severe CHD (excluded isolated ventricular septal defects, atrial septal defects, patent ductus arteriosus and pulmonic stenosis) and 264 control trios	Genes involved in production, removal and reading of methylation of H3K4 (H3K4me): *MML2*, *KDM6A*, *CHD7*, *WDR5*, *RNF20*, *UBE2B*, *SMAD2*	[44]
2871 CHD cases including parent-offspring trios and 1789 controls	*GDF1*, *MYH6*, *FLT4 CHD7*, *KMT2D*, *PTPN11*, *FLT4*, *NOTCH1*, *RBFOX2*, *SMAD6*, *GATA6*, *ELN*, *CCDC154*, *SLOCO1B3*, *GPBAR1*, *PTEN*, *RPL5*, *NSD1*, *SAMD11*, *C210RF2*, *NODAL*, *SMAD2*, *H1F00*, *FRYL*, *KDM5B*, *POGZ*, *SOS1*, *TBX18*	[41]
715 CHD parent-offspring trios and 416 healthy individuals	Mosaic *KMT2D* mutations	[46]
TOF, combined with Cleft Lip and Palate	G586A and G196S variant of *MTHFR*	[47]
8 TOF families	Novel causative mutations of *PEX5*, *NACA*, *ATXN2*, *CELA1*, *PCDHB4*, *CTBP1*	[48]
4 families with ASD, 2 families with PDA, 2 families with TOF and 1 family with dysplastic pulmonary valve	*GATA4* G115W (ASD), *TLL1* I263V (ASD kindred), *MYH11* (ductus arteriosus).	[49]
4-generation ASD family	Novel *GATA4* A899C, and K300T variants	[50]
79 subjects with BAV	*GATA4/5*, *APC*, *JAG1*, *NOTCH1/2/3*, *PAX8*, *SOX9*, *TBX5*, *WNT4*	[37]
Left-sided lesions	17 genes not previously associated with human cardiovascular malformation including *ACVR1*, *JARID2*, *NR2F2*, *PLRG1*, *SMURF1*. Gene established syndromic association with CHD such as *KMT2D*, *NF1*, *TBX20*, *ZEB2*.	[51]
181 individuals from 41 families with LVOTO	*NOTCH1*, *ARHGAP31*, *MAML1*, *SMARCA4*, *JARID2*, *JAG1.*	[40]
32 BAV patients	*NOTCH1* G4297A and putative pathogenic *ADMTS5* C935A.	[38]
Twins with heterotaxy	*MMP21*	[52]
1,365 trios with CHD, 68 probands from 32 multisibling families, and 458 singleton probands, 12,031 controls	*CHD4*, *CDK13*, *PRKD1*	[53]
59 CHD trios and 59 control trios, 100 affected singletons, 533 unaffected singletons	Novel *NR1D2* R175W mutation associated with AVSD, previously associated with CDH: *ZFPM2*, *NSD1*, *NOTCH1*, *VCAN*, and *MYH6*.	[54]
3 family members with LVNC	Novel genetic variant of *MYH7* gene	[55]

ASD: atrial septal defects; AVSD: atrioventricular septal defect; BAV: bicuspid aortic valve; LVNC: left ventricular non-compaction; LVOTO: left-ventricular outflow tract obstructions; TOF: tetralogy of Fallot.
